# C/EBP homologous protein promotes Sonic Hedgehog secretion from type II alveolar epithelial cells and activates Hedgehog signaling pathway of fibroblast in pulmonary fibrosis

**DOI:** 10.1186/s12931-022-02012-x

**Published:** 2022-04-08

**Authors:** Xiaoyu Yang, Wei Sun, Xiaoyan Jing, Qian Zhang, Hui Huang, Zuojun Xu

**Affiliations:** 1 Department of Respiratory and Critical Medicine, Peking Union Medical College Hospital, Chinese Academy of Medical Sciences and Peking Union Medical College, No. 1 Shuai Fu Yuan Street, Dong Cheng District, Beijing, 100730 China; 2 Medical Research Center, Peking Union Medical College Hospital, Chinese Academy of Medical Sciences and Peking Union Medical College, No. 1 Shuai Fu Yuan Street, Dong Cheng District, Beijing, China

**Keywords:** Pulmonary fibrosis, Type II alveolar epithelial cells, Fibroblast, CHOP, Shh

## Abstract

**Background:**

Endoplasmic reticulum (ER) stress is involved in the pathological process of pulmonary fibrosis, including IPF. It affects a broad scope of cellular types during pulmonary fibrosis but the role in epithelial-mesenchymal crosstalk has not been fully defined. The present study aimed to investigate the effects of Shh secretion by ER  stress-challenged type II alveolar epithelial cells (AECII) on fibroblast and pulmonary fibrosis.

**Methods:**

Conditioned medium (CM) from tunicamycin (TM)-treated AECII was collected and incubated with fibroblast. Short hairpin RNA (shRNA) was used for RNA interference of C/EBP homologous protein (CHOP). The effects of CHOP and HH signaling were evaluated by TM administration under the background of bleomycin-induced pulmonary fibrosis in mice.

**Results:**

Both expression of CHOP and Shh in AECII, and HH signaling in mesenchyme were upregulated in IPF lung. TM-induced Shh secretion from AECII activates HH signaling and promotes pro-fibrotic effects of fibroblast. Interfering CHOP expression reduced ER stress-induced Shh secretion and alleviated pulmonary fibrosis in mice.

**Conclusions:**

Our work identified a novel mechanism by which ER stress is involved in pulmonary fibrosis. Inhibition of ER stress or CHOP in epithelial cells alleviated pulmonary fibrosis by suppressing Shh/HH signaling pathway of fibroblasts.

**Supplementary Information:**

The online version contains supplementary material available at 10.1186/s12931-022-02012-x.

## Background

Despite the fact that people have increasingly acknowledged that idiopathic pulmonary fibrosis (IPF) is a fatal disease in recent decades, progress on its treatment has been slow, causing a huge burden on humanity [1, 2]. Even though the pathogenesis of IPF remains unclear, there is little doubt that this pathological illness involves sophisticated cellular interactions in the lung [3]. The crosstalk between epithelial cells and fibroblasts is the most well-studied of these pathways [4, 5]. Homeostasis loss of type II alveolar epithelial cells (AECII) is the initial factor for pulmonary fibrosis and subsequent development [6]. Various factors can lead to abnormal AECII function, such as infection [7], inhalation of toxic substances [8], and genetic susceptibility [9, 10]. Recently, endoplasmic reticulum (ER) stress was identified to leading AECII dysfunction and participated in the progression of pulmonary fibrosis, including IPF [11, 12]. ER stress is a cellular dysfunction caused by the accumulation of large amounts of incorrectly folded proteins in the ER. In order to relieve stress, cells recognize unfolded protein accumulation through receptors (PERK, ATF6, and Ire1) and initiate the unfolded protein response (UPR). UPR alleviates ER stress by promoting the expression of chaperone molecules and reducing protein translation, and if that fails, induces apoptosis. Among the effector molecules downstream of the UPR, the C/EBP homologous protein (CHOP) is a mutual effector transcription factor induced by all three UPR pathways and associated with the induction of apoptosis [13]. However, the mechanism by which ER stress-challenged AECII induced hyperactivation of mesenchymal cells, also known as aberrant epithelial-fibroblast cross talk, has still not been fully understood.

The morphogenetic signaling pathway Hedgehog (HH) signaling pathway was a vital pathway during lung development and maintenance of postnatal lung homeostasis [14]. Shh (Sonic Hedgehog), the most widely studied HH signaling pathway ligand, can be secreted by epithelial cells and acts on adjacent cell populations. In IPF, overexpression of Shh was revealed mainly in the alveolar epithelium [15, 16]. After Shh binds to the receptor PTCH, the downstream transcription factor glioma-associated oncogene homolog (GLI) family are transferred to the nucleus and regulate the transcription of the target gene [17]. The HH signaling responded mesenchymal Gli1^+^ cells were identified as critical contributors to organ fibrosis and form a pathological niche in the fibrotic lung [18]. Previous studies have demonstrated that the lung mesenchyme of adult mice retains a response to the HH signaling pathway, and most *Gli1*^+^ cells are fibroblasts [19]. In humans, the Hedgehog pathway also get reactivated in the IPF lung. IPF fibroblasts show overexpression of the main components of the Sonic Hedgehog signaling pathway, increased proliferation, Collagen and Fibronectin expression, and resistance to apoptosis [20]. Thus, the HH signaling has a significant role in controlling the malfunction of fibroblast. However, the mechanism regulating Shh production from AEC during pulmonary fibrosis is elusive.

In this study, we speculate that AECII secretes Shh under ER stress, subsequently activates fibroblasts in the mesenchyme, and thus participates in the abnormal repair process in pulmonary fibrosis. We also found that blocking CHOP expression could regulate ER stress-induced Shh expression and downstream fibroblast activation, which could be a potential target for the treatment of IPF.

## Materials and methods

### Human lung tissue

The use of samples in this study was approved by the Ethics Committee of Peking Union Medical College Hospital (JS-1127). IPF lung tissues (n = 6) were obtained from the department of lung transplantation, the First Affiliated Hospital of Guangzhou Medical University, Guangzhou, China. The IPF diagnoses were made by the guidance of the 2018 ATS/ERS/JRS/ALAT criteria [21]. Age and gender-matched non-fibrotic lung tissues (Additional file 1: Table S1) were obtained from 6 patients undergoing lung tumor resection in the thoracic surgery department of Peking Union Medical College Hospital and selected as non-fibrotic control.

### Cell line culture and treatment

Primary human AECII was purchased from Procell *Co. Ltd* (Wuhan, China) and cultured with DMEM/F12 (CORNING, 10% FBS, and 1% penicillin/streptomycin) in 37 ℃ and 5% CO_2_. Cultured human AECII had a round or polymorphic cell morphology and expressed AECII marker pro-SPC (Additional file 1: Fig S1). Human embryonic lung fibroblast MRC5 cells were purchased from the American Type Culture Collection (ATCC, Manassas, Virginia, USA). The cells were cultured in MEM (CORNING) with 1% non-essential amino acids (NEAA, gibco), 1% penicillin/streptomycin and 10% FBS at 37 ℃ under 5% CO_2_. The human Shh was purchased from Abcam (ab268966), tunicamycin from Sigma-Aldrich, and GANT61 from Selleckchem.

### Animal care and treatment

Animal experiments were approved by the Chinese Academy of Medical Science Laboratory Animal Center and conducted following the regulations established by the Institutional Committee for the Care and Use of Laboratory Animals. C57BL/6 mice (male, 6–8 weeks old) were purchased from Beijing Vital River laboratory animal technology *Co. Ltd*, and were maintained in Laboratory Animal Center, Peking Union Medical College Hospital. Mice were housed at a constant room temperature with a 12 h light/dark cycle, with free access to water and laboratory rodent food.

All treatment procedures for mice were based on endotracheal injection after anesthesia and described amply by others [22]. The lentivirus-packaged shRNA (1 × 10^8^ TU/mL, 100 μL/mouse) and tunicamycin (20 μg/mL in 100 μL of 20% DMSO diluted in PBS) were administered 2 days and 1 day before bleomycin injection, respectively. After 21 days post bleomycin injection, mice were sacrificed, and lungs were harvested for paraffin embedding, Western blot, and quantifying hydroxyproline.

### Enzyme-linked immunosorbent assay

The concentration of human Shh and TGFβ1 in AECII supernatant was measured by a commercially available ELISA kit: human Sonic Hedgehog ELISA kit (Abcam, ab100639) and human TGF beta 1 ELISA kit (Abcam, ab100647) according to the manufacturer’s instructions. The concentration of hydroxyproline was measured using Hydroxyproline Assay Kit (Colorimetric) (Abcam, ab222941) according to the manufacturer’s protocol.

### Western blot

Total proteins were collected by cell lysis with RIPA buffer with proteinase inhibitor on ice. Nuclear protein was prepared with cytoplasmic and nuclear extraction kits (Invent Biotechnologies) following the manufacturer’s instructions. The protein concentration was assessed using a BCA kit (ThermoFisher Scientific, Waltham, MA, USA). Protein samples were separated by 10% SDS-PAGE and transferred to a polyvinylidene fluoride (PVDF) membrane. The samples were incubated with primary antibodies overnight at 4 ℃. The membranes were incubated with horseradish peroxidase (HRP)-conjugated secondary antibody at room temperature for 1 h, and the immunoreactive protein bands were detected using a chemiluminescence device (GE, Amersham imager 680). The levels of protein expression were evaluated by measuring the gray value of the band by Fiji software, *n* = 3 in each group, and the relative value to β-actin was compared.

### Histology and immunofluorescence

Hematoxylin and eosin (HE), and Masson Trichrome staining were performed on paraffin-embedded tissue sections. HE stained sections were used for Ashcroft scoring. Ashcroft score was performed by averaging the scores from one blinded and one non-blinded scorer. The fibrotic areas in lung tissue were quantified using Fiji software.

Lung tissue sections were deparaffined with xylene for 20 min twice and rehydrated in gradient reduced ethanol (100% twice, 95%, 90%, 75%, and 50% once) for 5 min each. Antigen retrieval was performed for 30 min at 95 ℃ using 0.01 M sodium citrate buffer (Solarbio, pH 6.0). The sections were then blocked for 1 h with 5% FBS in PBST at room temperature. For human lung tissue, the slides were incubated with primary antibodies as follows: rabbit anti-prosurfactant protein C antibody (Abcam, ab90716), mouse anti-CHOP (DDIT3) antibody (Abcam, ab11419), mouse anti-Sonic Hedgehog antibody (Abcam, ab135240), rabbit anti-Sonic Hedgehog (Abcam, ab73958), rabbit anti-alpha smooth muscle Actin antibody (Abcam, ab5694), mouse anti-alpha smooth muscle Actin antibody (Abcam, ab7817), rabbit anti-Gli1 antibody (Abcam, ab134906), rabbit anti-Gli2 antibody (Abcam, ab277800), rabbit IgG (Abcam, ab172730) and mouse IgG (Abcam, ab37355). For mouse lung tissue, the primary antibodies were mouse anti-Sonic Hedgehog antibody (Abcam, ab135240) and rabbit anti-prosurfactant protein C antibody (Abcam, ab211326). They were then incubated overnight at 4 ℃. The secondary antibodies were Alexa Fluor 488-conjugated goat anti-rabbit IgG antibody (Jackson ImmunoResearch) and Alexa Fluor 594-conjugated goat anti-mouse IgG antibody (Jackson ImmunoResearch), incubated for 1 h at room temperature.

### Immunofluorescence images quantification

Fluorescence labeled slides for lung tissue and cultured cells are analyzed by confocal microscopy (Nikon). For the relative quantification of the expression levels of CHOP and Shh in AECII of IPF patients, we randomly selected 10 fields (200 ×) of view and calculated the mean fluorescence intensity of the red channels in the red and green colocalized regions using Fiji software, normalized with the mean value of the normal group. In order to quantify the expression of Shh and CHOP in alveolar epithelial cells of human lung tissues, we randomly selected 10 fields (200 ×) and counted the epithelial cells in each field that were positive for both Shh and CHOP. The selected region from IPF include normal alveolar structures, including alveoli wall and lumen to distinguish AEC by microscopic anatomical structures. For quantification of protein expression of AECII and MRC5, 10 fields were randomly selected, and the percentage of pixel intensity for each RGB channel was analyzed with Fiji software. Cell counts were performed by automatically calculating for DAPI by Fiji software using the Analyze Particles tool. For the relative quantification of the Shh positive AECII in AECII of mice, we randomly selected 10 fields (200 ×) of view, the number of Shh positive AECII was manually counted by two separate counters. At least 3 animals per group were used.

### RNA interference

Lentivirus-packaged shRNA was purchased from GenePharma *Co. Ltd* for in vitro and in vivo experiments, and a scrambled shRNA (NC) act as the control. All vectors carried puromycin resistance genes for screening. Primary human AECII was seeded in a 6-well plate and replenished with a medium containing 1 × 10^7^ TU/mL lentivirus and 5 μg/mL polybrene. After incubating for 24 h, replenish AECII with fresh DMEM/F12 complete medium and continue culture for 48 h. Untransfected AECII can be screened by puromycin (10 μg/mL). The intervention efficiency was tested in primary AECII (Additional file 1: Fig S2) and mouse epithelial cells in vivo.

### Collection of AECII conditioned medium (CM)

Primary human AECII was maintained in DMEM/F12 until it reached 90% confluence in a 10 cm dish. Then, TM was added with the concentration of 5 μg/mL and incubated for 48 h. After that, the medium was discarded and washed with PBS. The washed AECII was replenished with 10 mL serum-free DMEM/F12 and continued to culture for 72 h before harvest. The harvested media was collected and centrifuged at 500*g* for 10 min to remove cell debris. Supernatants were then filtered through a 0.22 μm filter. Finally, the filtered medium was concentrated with a 3 kDa cutoff centrifugal filter unit (Millipore) by a factor of 50. Collected CM kept at – 80 ℃ until use.

### MRC5 proliferation assay

Subconfluent MRC5 were cultured in reduced FBS (2.5%) for 48 h in the presence of blank MEM, TM-CM, and TM/sh*CHOP*-CM, respectively, on 96-well plates with clear bottom. For the expression of Ki67, MRC5 cells were incubated with FITC conjugated anti-Ki-67 antibody (Invitrogen, 7B11) after fixation and permeabilization (eBioscience, 00-5521-00) and measured by BD FACSCelasta flowcytometry. For the BrdU incorporation assay, cells were labeled with BrdU (luminometric BrdU cell proliferation ELISA; Roche). Finally, samples were read on absorbance from 370 to 492 nm using SpectraMax Gemini EM (Sunnyvale, CA, USA).

### Flow cytometry and sorting for RNA isolation

Mice lungs were finely cut and digested with 200 U/mL type 1 collagenase (gibco), 2 U/mL dispase 1 (Sigma-Aldrich) in HBSS (with Ca^2+^ and Mg^2+^) in the condition of 37 ℃ water bath for 1 h. Single-cell suspensions were harvested by passing through a 70-μm mesh filter and RBC lysis. The epithelial cells were labeled with PE-conjugated anti-EpCAM antibody (Abcam, ab237387) and sorted with BD FACSAria II flowcytometry using FACSDiva software.

### RNA purification and qPCR

Total RNA was obtained from cultured primary human AECII, MRC5, and sorted epithelial cells using RNeasy kit (Qiagen, 74,004) following the manufacturers’ instructions. extracted and followed by reverse transcription into complementary DNA with the ThermoScript reverse transcription PCR system (Invitrogen, CA, USA) for the subsequent qPCR analysis. qPCR was performed using SYBR Green system (ThermoFisher), and results were analyzed after 40 cycles of amplification using ABI 7500 fast real-time PCR system. Relative expression levels of genes were defined by ∆∆Ct method and normalizing to β-actin. The primer sequences were listed in Additional file 1: Table S3.

### MTT assay

AECII (2.5 × 10^5^ cells/mL) were seeded into a 96-well plate. Then cells were maintained in DMEM/F12 with or without TM in the incubator at 5% CO_2_ and 37 °C. The plate was removed on days 0.5, 1, 2, 3, and 4 for the MTT assay. Briefly, 10µL MTT solution (5 g/L) (Solarbio) was added to each well. Cells were then further incubated at 37 °C for 24 h. This was followed by the addition of 100 µl DMSO. The plate was then slightly shaken and mixed evenly for 10 min. An automatic enzyme-labeled reading meter (Thermo) was used to measure the optical density value at 490 nm.

### Statistical analysis

All the values are shown as mean ± SD, and the comparison of mean value between two groups using two-tailed *t* test (parametric analysis for *n* > 3 or nonparametric analysis for *n* = 3). Survival analysis was conducted with the Kaplan–Meier method and log-rank tests. One-way ANOVA was used for the mean differences between three groups and Tuckey’s multiple comparison test for comparison among each group with Prism GraphPad 9.0.0 software. Significance level is indicated by *P < 0.05, **P < 0.01, ***P < 0.001 and ****P < 0.0001.

## Results

### IPF AECII suffer ER stress and excessive Shh production, along with Hedgehog activation in the mesenchyme

**Fig. 1:**
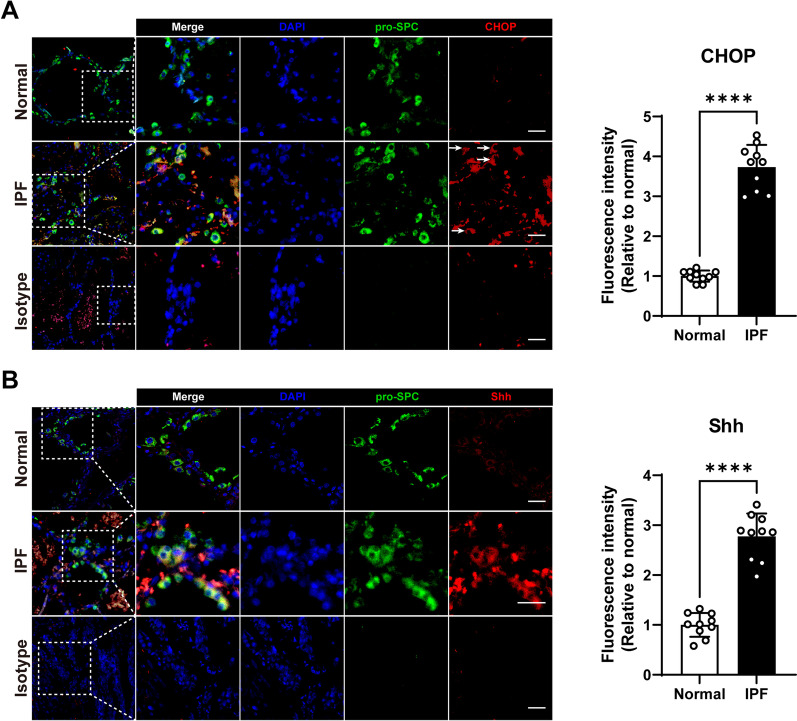
Detection of CHOP and Shh in AECII in normal and IPF lung tissue. **A** Representative immunofluorescence images on paraffin section by dual staining for pro-SPC (green) and CHOP (red) of normal (*n* = 6) and IPF (*n* = 6) lung tissue. Arrows indicate CHOP positive AECII. **B** Representative immunofluorescence images on paraffin section by dual staining for pro-SPC (green) and Shh (red) of normal (*n* = 6) and IPF (*n* = 6) lung tissue. Bar = 20 μm

**Fig. 2:**
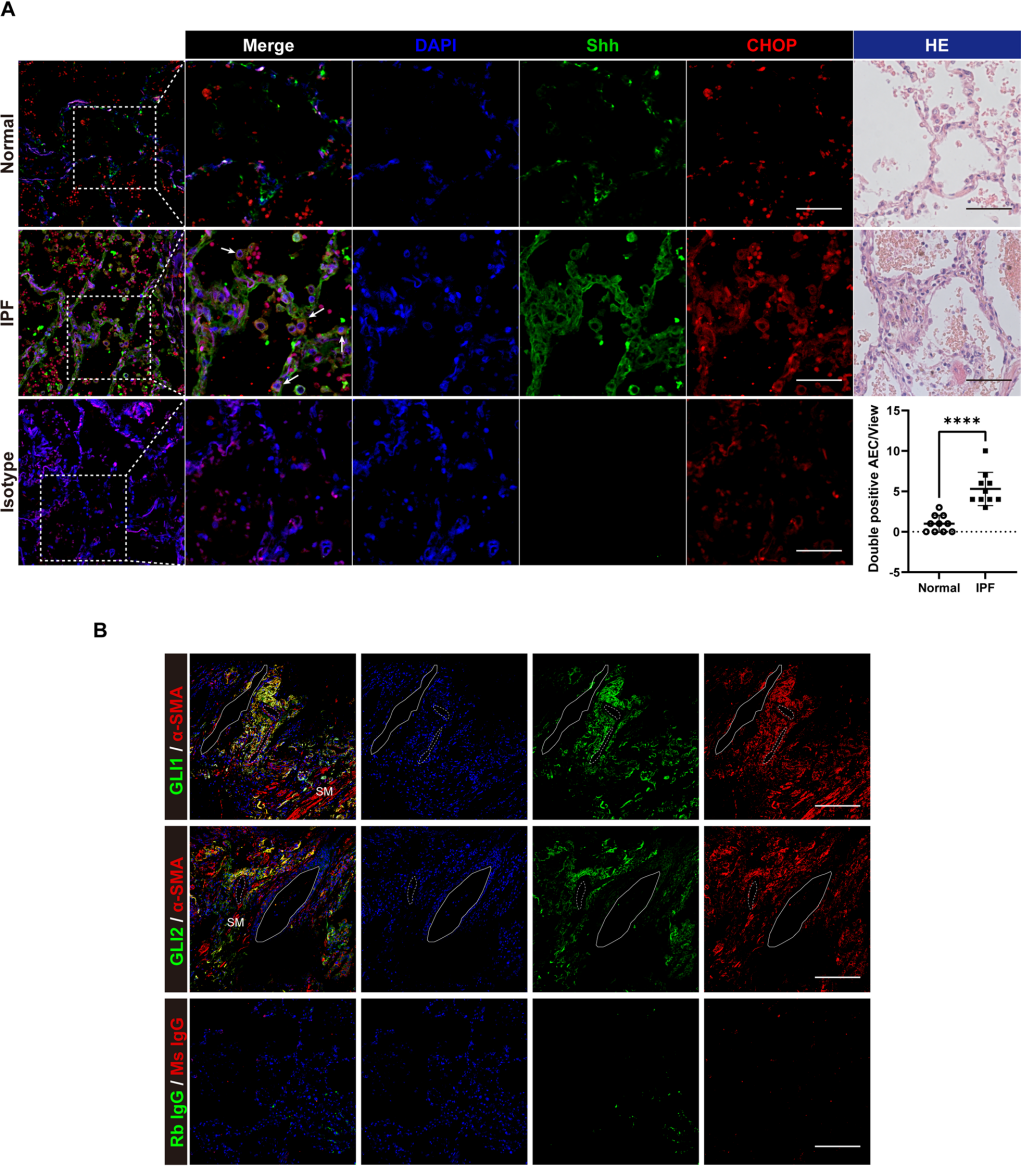
IPF lungs show extensive HH signaling activation. **A** Representative immunofluorescence and HE images on paraffin section by dual staining for Shh (green) and CHOP (red) of normal (*n* = 6) and IPF (*n* = 6) lung tissue. Arrows indicate AECII expressing CHOP and Shh. Bar = 20 μm. **B** Detecting the GLI1 and GLI2 expression patterns in IPF lungs show prominent expression of both GLI1 and GLI2 in lung mesenchyme. α-SMA (red) positive cells indicate smooth muscle outline airways and vessels, as well as myofibroblasts. Solid lines, airways. Dotted lines, blood vessels. SM, smooth muscles. Bar = 50 μm

Various stimuli incite ER stress. As a result, cells undergo unfolded protein response (UPR), which increases CHOP expression. To explore the effect of endoplasmic reticulum stress on epithelial cells during IPF, we performed immunofluorescence assays on lung tissue from IPF patients. HE and Masson staining of lung tissue from IPF patients show disruption of alveolar structure and accumulation of extracellular matrix (Additional file 1: Fig. S3). In normal lung tissues, the expression of CHOP is sparse. While in IPF lung tissue, the expression of CHOP was elevated (Additional file 1: Fig S4). We confirmed that extensive ER stress occurred in epithelial cells during IPF by elevated CHOP expression in pro-SPC-labeled AECII (Fig. 1A). The expression of Shh in AECII in IPF lung also increased (Fig. 1B). These phenotypes suggest that ER stress and increased Shh synthesis in AECII may involve IPF processes. We further found that CHOP and Shh colocalized in the IPF AECII (Fig. 2A), suggesting a close link between the two biological effects. The HH signaling maintains the homeostasis of lung cells among epithelium and mesenchyme. In IPF lung, HH signaling downstream transcriptive factors Gli1 and Gli2 were expressed universally in the lung mesenchyme (Fig. 2B), indicating widely activated HH signaling. However, the relationship between these two biological processes remains unclear.

### Interfering CHOP expression downregulates ER stress-induced Shh overexpression

**Fig. 3:**
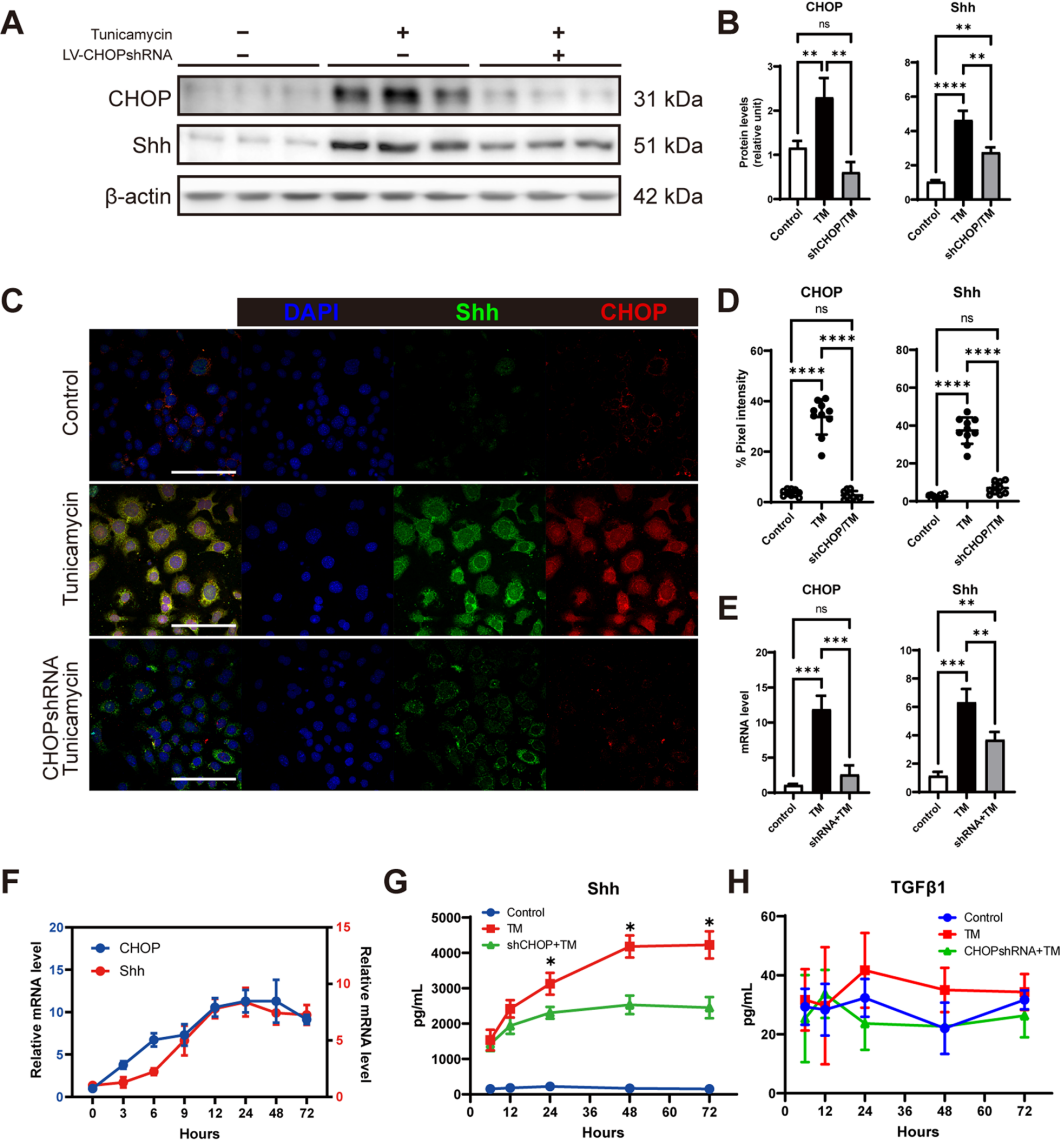
Interfering *CHOP* during ER stress reduces Shh secretion of AECII. **A** Expression of CHOP and Shh were examined by Western blotting. β-actin was used as the loading control. Each group has 3 replicates. **B** Quantification of expression by measuring relative band gray value of blotting which normalized by β-actin. ns, non-significant. **C** Representative immunofluorescence images on treated MRC5. The experiment was repeated three times. Bar = 50 μm. **D** Quantification of percent pixel intensity of CHOP and Shh. Each dot represents data from one field; *n* = 10 independent measures. ns, non-significant. **E** mRNA level of CHOP and Shh relative to control (24 h post-treatment). **F** Kinetic measurements of the mRNA level of CHOP and Shh in AECII. (G-H) ELISA for Shh and TGFβ1 in the supernatant of AECII. Differences in the mean between TM and sh*CHOP*/TM treatment groups were compared

To investigate the relationship between ER stress and Shh overexpression, we used tunicamycin (TM, 1 μg/mL) to treat primary human AECII. In this TM concentration, AEC did not show significant apoptosis within 24 h (Additional file 1: Fig. S4) but induced CHOP expression (Fig. 3A, B). TM treatment also caused an elevated level of Shh (Fig. 3A, B), which indicates the upregulation of Shh in the condition of ER stress. To block the biological effects downstream of CHOP during ER stress, we constructed lentiviral-packaged shRNA, specifically silencing *CHOP* (sh*CHOP*) and transfected AECII. The sh*CHOP* successfully silenced CHOP expression in AECII and suppressed TM-induced Shh overexpression (Fig. 3A, B). In addition, we confirmed the above effect utilizing immunofluorescence (Fig. 3C, D) and qPCR (Fig. 3E). By kinetic observation of the mRNA level of CHOP and Shh after TM treatment, we found that the transcriptional elevation time point of *CHOP* was earlier than that of *Shh* (Fig. 3F), which indicates that the CHOP upregulation regulated the expression of Shh. Shh is synthesized intracellularly and secreted extracellularly to affect adjacent cells as a ligand for the HH signaling pathway. Therefore, we detected the concentration of Shh in the cell culture medium by ELISA (Fig. 3G). Consistent with intracellular Shh expression levels, TM promotes Shh secretion, which can peak after 48 h. Shh secretion is reduced after silencing of *CHOP*. Moreover, we did not detect significant secretion of pro-fibrotic growth factor TGFβ1 in the AECII medium (Fig. 3H).

### *CHOP* silence of AECII suppressed Shh/HH signaling activation of lung fibroblast

**Fig. 4:**
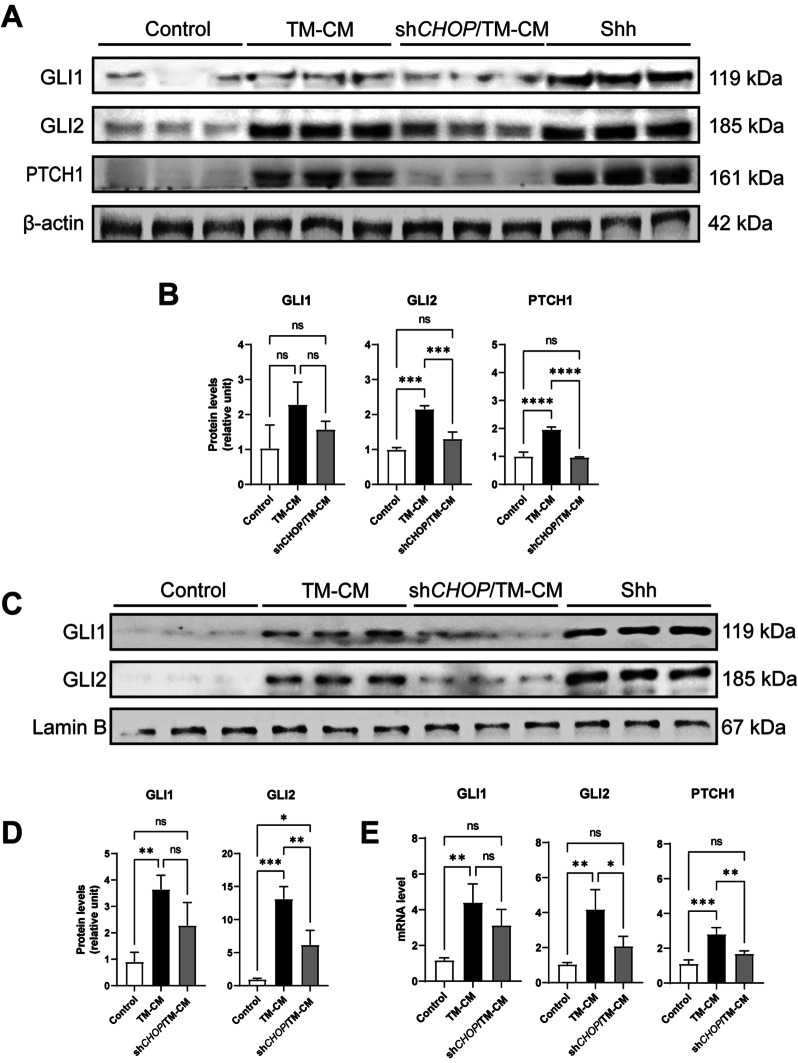
Interfering *CHOP* of AECII abrogates HH activation of fibroblast. MRC5 was incubated with AECII CM for 72 h. **A** Expression of GLI1, GLI2, and PTCH1 was examined by Western blotting. Shh (100 ng/mL) was used as the positive control. β-actin was used as the loading control. Each group has 3 replicates. **B** Quantification of protein level relative to control. **C** Expression of nuclear GLI1 and GLI2 by Western blotting. Lamin B was used as the loading control. **D** Quantification of protein level of nuclear GLI1 and GLI2 relative to control. **E** mRNA levels of GLI1, GLI2, and PTCH1 in MRC5 relative to control

To further study the effect of AECII Shh secretion regulated by CHOP on fibroblasts, we collected CM from cultured AECII treated without or with *CHOP*shRNA transfection before TM treatment (TM-CM and sh*CHOP*/TM-CM, respectively). Human fibroblast cell line MRC5 cultured with TM-CM for 72 h showed a significantly elevated level of transcription factors GLI1, GLI2, and receptor PTCH1 in both protein expression (Fig. 4A, B) and mRNA transcription (Fig. 4E), indicating activation of Hedgehog signaling pathway of MRC5. In addition, after Shh ligand binding with Smo, cytoplasmic GLI family proteins translocate to the nucleus and regulate target genes transcription. As expected, TM-CM cultured MRC5 shows more GLI1 and GLI2 nuclear translocation (Fig. 4C). However, the activation of HH signaling was not apparent when MRC5 was cultured with sh*CHOP*/TM-CM, except for GLI2 nuclear translocation (Fig. 4C, D).

### *CHOP* silence of AECII inhibits pro-fibrotic phenotype and proliferation of lung fibroblast

**Fig. 5:**
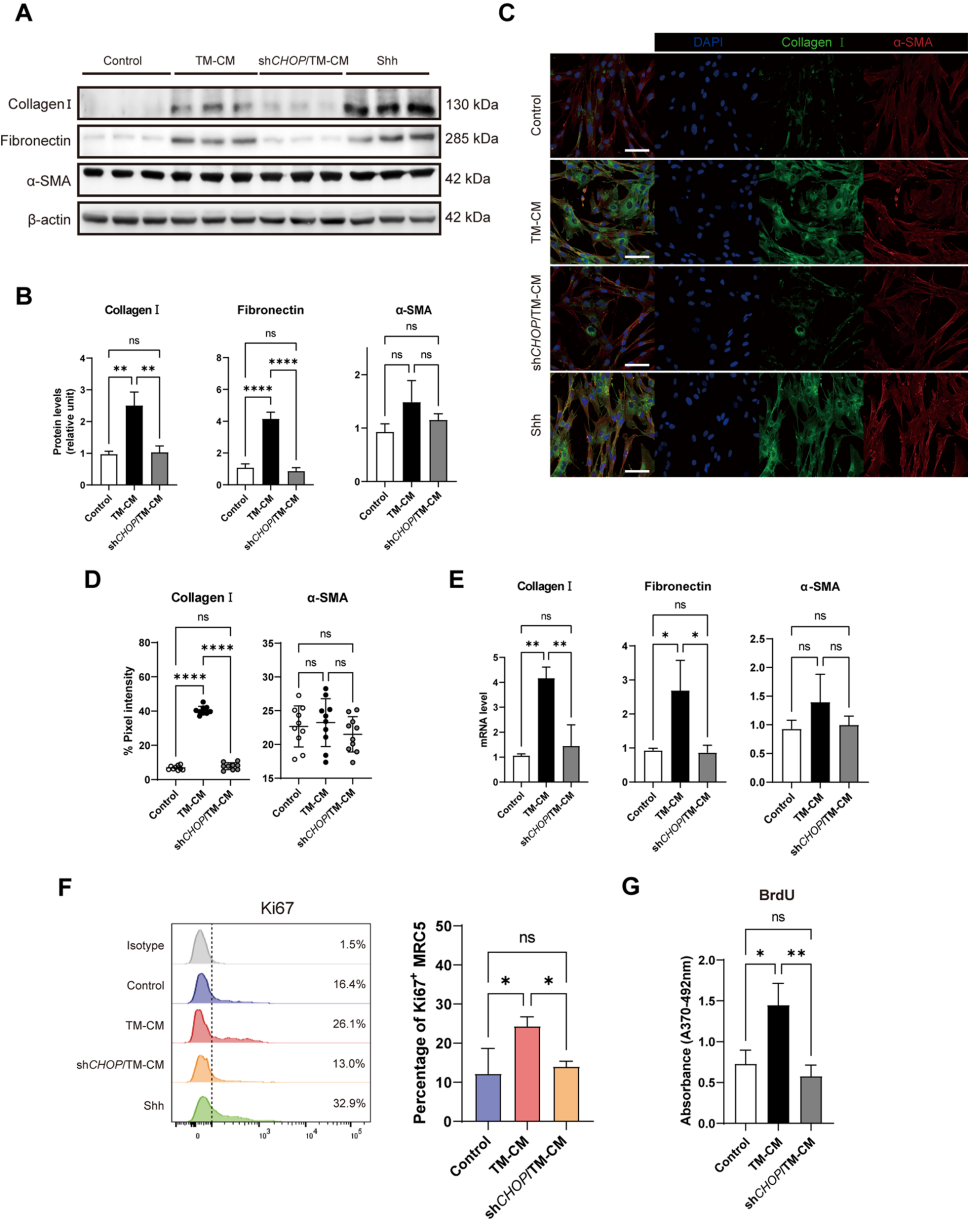
Interfering *CHOP* of AECII abrogates the pro-fibrotic effect of fibroblast. MRC5 was incubated with AECII CM for 72 h. **A** The expression of Collagen I, Fibronectin, α-SMA were examined by Western blotting. Shh (100 ng/mL) was used as the positive control. β-actin was used as the loading control. **B** Quantification of protein levels relative to control. **C** Representative immunofluorescence images on CM treated MRC5 by dual staining of Collagen I (green) and α-SMA (red). The experiment was repeated three times. Bar = 20 μm. **D** Quantification of percent pixel intensity of Collagen I and α-SMA. Each dot represents data from one field; *n* = 10 independent measures. **E** mRNA level of Collagen I, Fibronectin, and α-SMA relative to control. **F** The proliferation of CM treated MRC5 was examined by the percentage of Ki67 positive cells. Shh (100 ng/mL) was used as the positive control. **G** The proliferation of CM-treated MRC5 was examined by BrdU incorporation assay

Activation of the HH signaling pathway was reported to promote pro-fibrotic characteristics of fibroblasts. Our study found that 100 ng/mL Shh induced sufficient fibroblast activation to express more extracellular matrix components such as Collagen I and Fibronectin. However, the myofibroblast hall marker α-SMA was not significantly increased (Fig. 5A, C). Then, we evaluated the pro-fibrotic effect of TM-CM and sh*CHOP*/TM-CM on fibroblast by Western blot, immunofluorescence, and qPCR. We found that only TM-CM can stimulate MRC5 to increase collagen I and fibronectin expression but not sh*CHOP*/TM-CM (Fig. 5A–E). Neither CM changed the α-SMA level of MRC5. We also found that TM-CM promotes MRC5 proliferation, but sh*CHOP*/TM-CM did not promote proliferation by flowcytometric analysis for Ki67 positive MRC5 (Fig. 5F) and BrdU incorporation (Fig. 5G).

### Suppression of *Chop* in the lung ameliorate pulmonary fibrosis induced by ER stress

**Fig. 6:**
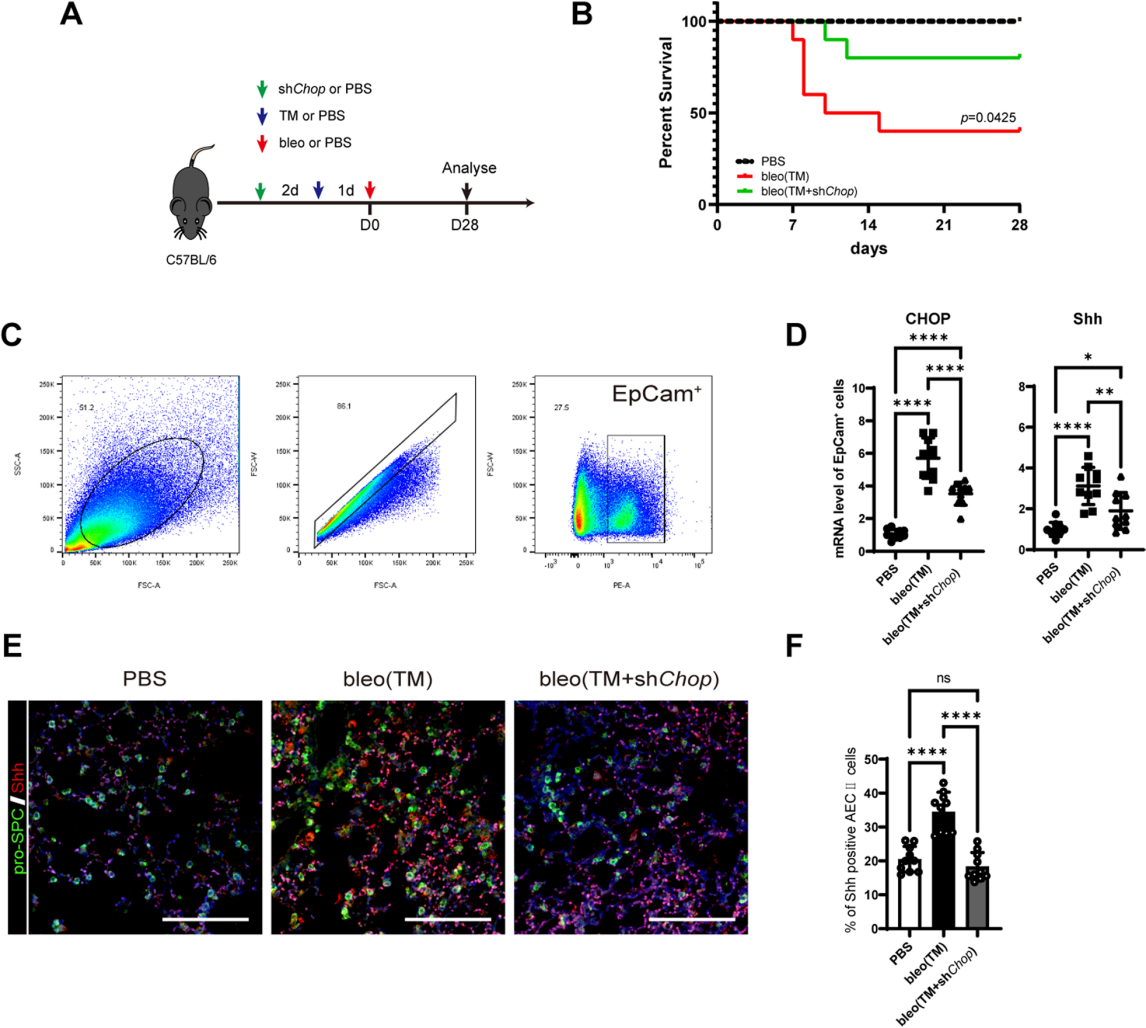
LV-*Chop*-shRNA promotes survival of tunicamycin aggravated pulmonary fibrosis by bleomycin. **A** Schematic of the experimental study in C57BL/6 mice. sh*Chop*, LV-*Chop*-shRNA; TM, tunicamycin; bleo, bleomycin. **B** Kaplan–Meier analysis for TM/bleo mice treated with or without sh*CHOP*. *n* = 10 biological independent animals per group. **C** Gating strategy for flowcytometric sorting of EpCAM positive epithelial cells of mouse lung. **D** mRNA level of CHOP and Shh of sorted epithelial cells relative to control. Each dot represents cells from an independent mouse. *n* = 10 per group. **E** Representative immunofluorescence images on mouse lung. AECII was labeled with pro-SPC (green) and Shh (red). Bar = 50 μm. **F** Quantification of the percentage of Shh positive AECII in mouse lung. Each dot represents cell numbers in one field. *n* = 10 per group

ER stress has been shown to accentuate pulmonary fibrosis in mice. Based on the above findings, we conducted in vivo experiments to investigate the effects of ER stress and the HH signaling pathway in the bleomycin-induced mice model of pulmonary fibrosis. ER stress was induced by endotracheal injection of TM (2 μg/20 g body weight) 1 day before bleomycin injection, and pulmonary fibrosis was prominent in 21 days post bleomycin injection. In addition, we suppressed CHOP expression in lung epithelial cells by endotracheal instillation of lentiviral-packaged shRNA, which specifically interfered CHOP expression (Fig. 6A). We found that sh*Chop* significantly improved the survival rate during induction of the pulmonary fibrosis with TM followed by bleomycin (Fig. 6B). This phenomenon encourages us to explore further whether the alleviating effect of sh*Chop* on pulmonary fibrosis is due to the mechanism in vitro. We next examined whether sh*Chop* injection affected CHOP expression in the epithelium. *Chop* silencing efficiency was verified 8 days after TM (7 days after bleomycin) endotracheal injection by qPCR to detect CHOP mRNA levels in EpCAM-positive cells sorted by flow cytometry (Fig. 6C). As with in vitro effects, TM installation increased Shh mRNA level in the lung epithelial cells (Fig. 6D) and Shh positive AEC II (Fig. 6E, F), while sh*Chop* pretreatment abolished these effects.

**Fig. 7:**
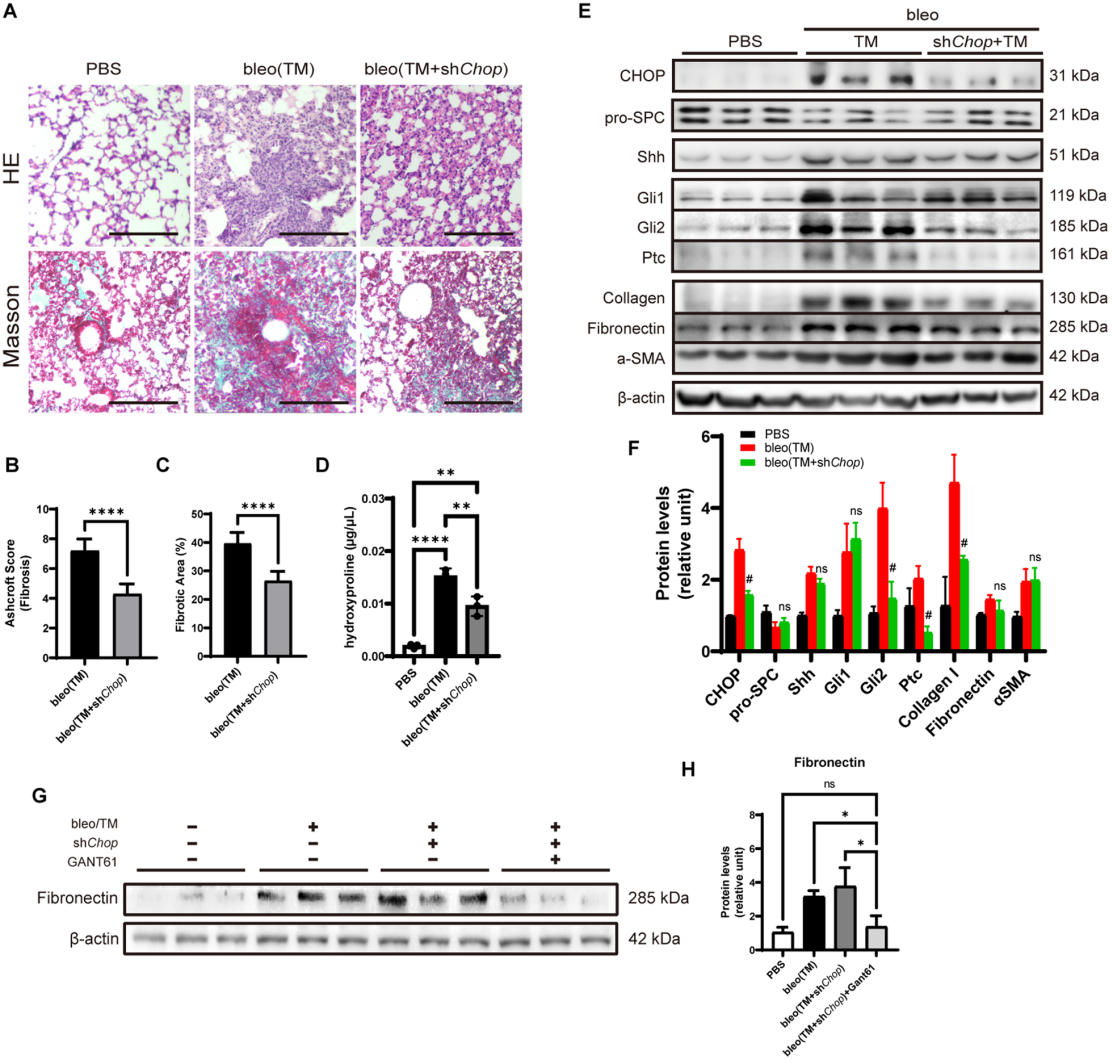
LV-*Chop*-shRNA reduced pulmonary fibrosis and fibroblast activation. **A** Pulmonary fibrosis was determined by hematoxylin–eosin (H&E) staining and Masson’s trichrome staining. Representative images of three independent experiments are shown. Bar = 100 μm. **B** Quantification of pulmonary fibrosis by Ashcroft score. **C** Quantification of pulmonary fibrosis by measuring fibrotic area in lung sections with Masson’s trichrome staining. **D** Quantification of pulmonary fibrosis by measuring hydroxyproline of mouse lung. **E** Expression of proteins involved in ER stress, HH signaling pathway, and fibroblast activation was examined with Western blotting. β-actin was used as the loading control. **F** Quantification of protein levels relative to control. # indicates significance in the comparison between bleo(TM + sh*Chop*) and bleo(TM) group. ns, non-significant. **G** Expression of Fibronectin after adding GANT61 in mouse lung. (H) Quantification of protein levels relative to control

The sh*Chop* pretreatment significantly ameliorates TM-induced Pulmonary fibrosis by HE (Fig. 7A, B), Masson staining (Fig. 7A, C), and hydroxyproline content (Fig. 7D). Moreover, we found that the Shh, Gli1, Gli2, and Ptc expression increased, and Shh, Gli2, and Ptc downregulated after silencing *Chop* in the lung (Fig. 7E, F). TM induction also increases Collagen I and Fibronectin expression in the lung, as well as α-SMA. sh*Chop* reduced Collagen I accumulation by Western blot but failed in Fibronectin (Fig. 7E, F). We speculate that the reason for this phenomenon is that the HH signaling pathway is not sufficiently blocked (considering that Gli1 still has a higher level than the control lung). Therefore, we evaluated the effect of Gli1/Gli2 inhibitors GANT61 on model mice. Supplementation with GANT61 (25 mg/kg) reduced the level of Fibronectin by Western blot compared with sh*Chop*/TM treatment (Fig. 7G, H), indicating a significant alleviating on pulmonary fibrosis.

## Discussion

IPF is a fibrotic lung disease that can result in irreversible lung remodeling. However, because the pathogenic etiology of IPF is unknown, there is no viable treatment for end-stage cases other than lung transplantation [23, 24]. According to current understanding, the main factor that causes and facilitates the progression of IPF is the loss of stability and injury to the AEC [25, 26]. When AEC is challenged to stress, they release a variety of factors that build a pathological niche and influence the behavior of adjacent cells, including fibroblasts. That may explain why antifibrotic medicine (Pirfenidone and Nintedanib) that targets pro-fibrotic signaling pathways slows lung function decrease but does not appreciably extend survival time by failing to rescue the AEC from stress [27, 28].

In recent decades, ER stress has been identified to be involved in IPF by affecting AECII. AECII has a developed endoplasmic reticulum system and is vulnerable to ER stress as a type of epithelium with robust secretory function and proliferation capabilities [6]. Studies on ER stress affecting IPF is mainly centered on ER stress-mediated AECII apoptosis. Korfei et al. discovered that the expression of major UPR-associated molecules was upregulated in lung tissues of IPF patients, particularly in AEC, which was the first evidence of ER stress-mediated apoptosis in AEC in sporadic IPF [29]. Since then, many studies on the mechanisms of ER stress-induced apoptosis in AECII have emerged, including gene mutation, viral infection [7], and hypoxia [30]. However, it appears that the effects of ER stress on AEC in causing pulmonary fibrosis are not confined to initiating apoptosis, and investigations have proven the multiple impacts of ER stress on pulmonary fibrosis. ER stress impairs mitochondrial homeostasis of AEC by repressing PINK1 transcription [31]. In addition, chaperone defects induced ER stress aggravates the progression of pulmonary fibrosis by inducing AECII senescence, which is consistent with the characteristics of IPF patients who are mainly elderly [32]. In addition, when the AECII is damaged, they initiate their proliferation to rebuild the epithelium. In the IPF, a considerable quantity of AECII proliferation or AEC hyperplasia can be observed. In this condition, AECII may also be under ER stress and start UPR activation, with unfolded protein accumulation in the ER to fulfill the increased metabolic demand. In this work, tunicamycin combined bleomycin treatment reduced pro-SPC levels in mice, while sh*Chop* pretreatment ameliorated fibrosis. However, the pro-SPC levels failed to recover to the basal level after (Fig. 6E, F), indicating that ER stress/CHOP influences pulmonary fibrosis through an alternative mechanism other than AECII apoptosis.

After AEC injury, aberrant epithelial-fibroblast interactions during lung healing triggers lung fibrosis. The HH signaling pathway maintains epithelium and mesenchyme relatively stable in both normal and injured situations [33]. However, in IPF patients, the HH signaling pathway is over-activated and is thought to be involved in the pathological process of IPF. In terms of mechanism, the HH signaling pathway upregulates a large amount of extracellular matrix (ECM) synthesis, mainly through the activation of fibroblasts. Shh treatment promotes fibroblast proliferation, survival, migration, and ECM production without myofibroblast differentiation (elevating α-SMA expression) [20]. Our in vitro results (Fig. 3A–E) are consistent with this study, and this phenomenon suggests fibroblast activation induced by the Shh/HH signaling pathway, unlike other pathways such as TGFβ/SMAD signaling does not lead to the transformation of fibroblasts to myofibroblasts. HH signaling also controls fibroblast activation and tissue fibrosis in systemic sclerosis, a disease in which interstitial lung disease (ILD) and fibrosis are common pulmonary complications [34]. Inhibition of the HH pathway at GLI transcriptive activity level reduced bleomycin-induced mouse pulmonary fibrosis [35]. In our study, the proliferated mesenchyme shows a prominent expression of GLI1 and GLI2 in IPF, indicating HH signaling activation. Activation of the HH signaling pathway is dependent on the binding of Hh ligands to the receptors on the cell membrane. AECII is the primary cellular source of Shh during IPF [16, 36, 37]. In this study, we also observed Shh expression of AECII in IPF lung (Fig. 1B). However, the factors responsible for the upregulation of Shh in AECII of IPF are not identified.

Recently, ER stress was reported inducing the sonic HH pathway in diabetic liver injury in mice [38]. CHOP, a crucial transcription factor associated with ER stress, can be induced to be upregulated only in response to ER stress-associated UPR. Its expression can be regulated by all three branchings of UPR (PERK, ATF6, and IRE1 recognized), making it a marker with reasonable specificity and sensitivity [39]. Klymenko et al. demonstrated that overexpression of active ER stress sensors did not induce *Chop* transcription in mouse AECII [40], which is the reason we did not examine the expression of these sensors in this study. This study found significantly elevated CHOP expression in AECII in IPF lung tissue (Fig. 1A), consistent with previous studies [30]. In addition to apoptosis induction, CHOP is also a multifunctional transcription factor [41], and the downstream biological effects of CHOP are not yet fully understood. For example, CHOP is crucial for the induction of caspase-11 and promotes the processing of pro-IL1β in LPS induced inflammation [42]. However, current studies have primarily confirmed that inhibition of CHOP plays a protective role in the process of pulmonary fibrosis.

Interestingly, we also observed the colocalization of Shh and CHOP in AECII of IPF lung (Fig. 2A). Therefore, we speculated that ER stress-induced CHOP might be involved in the regulation of Shh. By treating primary human AECII with ER stress inducer tunicamycin, we found upregulation and secretion of Shh. In addition, this ER stress-induced Shh upregulation and the pro-fibrotic effects on fibroblast can be depleted by interfering with CHOP expression. In vivo, we used sequential injections of tunicamycin and bleomycin to construct a pulmonary fibrosis model because tunicamycin-induced ER stress alone did not induce sufficient pulmonary fibrosis [43]. To assess the function of CHOP, we used shRNA from a lentiviral vector to interfere with epithelial CHOP expression via intratracheal injection. Although inhibition of CHOP improved pathological manifestations of pulmonary fibrosis, reduced hydroxyproline content, and level of Collagen I, no significant reduction in Fibronectin was observed (Fig. 6E). We speculated that the reasons might be as follows: 1. The intervention utilizing lentiviral vector transfection is not efficient enough, and therefore, *Chop* gene conditional-knockout mice is an urgent need for our following experiments; 2. Shh is abundantly sourced, and the way to interfere with *Chop* in epithelial cells alone does not sufficiently interfere with Shh, and our experiments also show that the decrease in Shh is not significant in sh*Chop* treated lung homogenates; 3. The production of Shh involves many unrecognized regulatory modalities, and there are still other ways to promote Shh expression after simple interference with *Chop*. Our results also showed that Gli1 remained at high levels after interfering with CHOP expression. However, adding a GLI family DNA binding inhibitor, GANT61 [44], resulted in a significant decrease in Fibronectin, demonstrating the critical effect of HH signaling in alleviating pulmonary fibrosis exacerbated by ER stress. Although in vitro TM induction did not upregulate TGFβ1 secretion from AECII (Fig. 3H), the effect of TGFβ could not exclude in the bleomycin model.

Although we confirmed the relationship between ER stress and Shh secretion of AECII in detail, there are still some limitations in this study. First, AECII and fibroblasts derived from patients with IPF appear to be more representative of pathological conditions in vitro. ER stress induction by tunicamycin may exaggerate the in vivo role of ER stress. Second, our current results failed to elucidate the molecular mechanism of Shh regulation by CHOP. The absence of Shh leads to ER stress in mouse intestinal epithelial cells [45], and the HH signaling pathway as well as ER stress were activated in the liver of mouse model of type 1 diabetes [38]. Given that HH signaling pathway is essential for cell growth and regeneration, we speculate that ER stress cause cells to secrete the HH ligand Shh to promote epithelial regeneration for compensation. Over-secretion of Shh to promote fibroblast activation and proliferation is an unintended byproduct of stress-challenged AECII.

## Conclusion

**Fig. 8:**
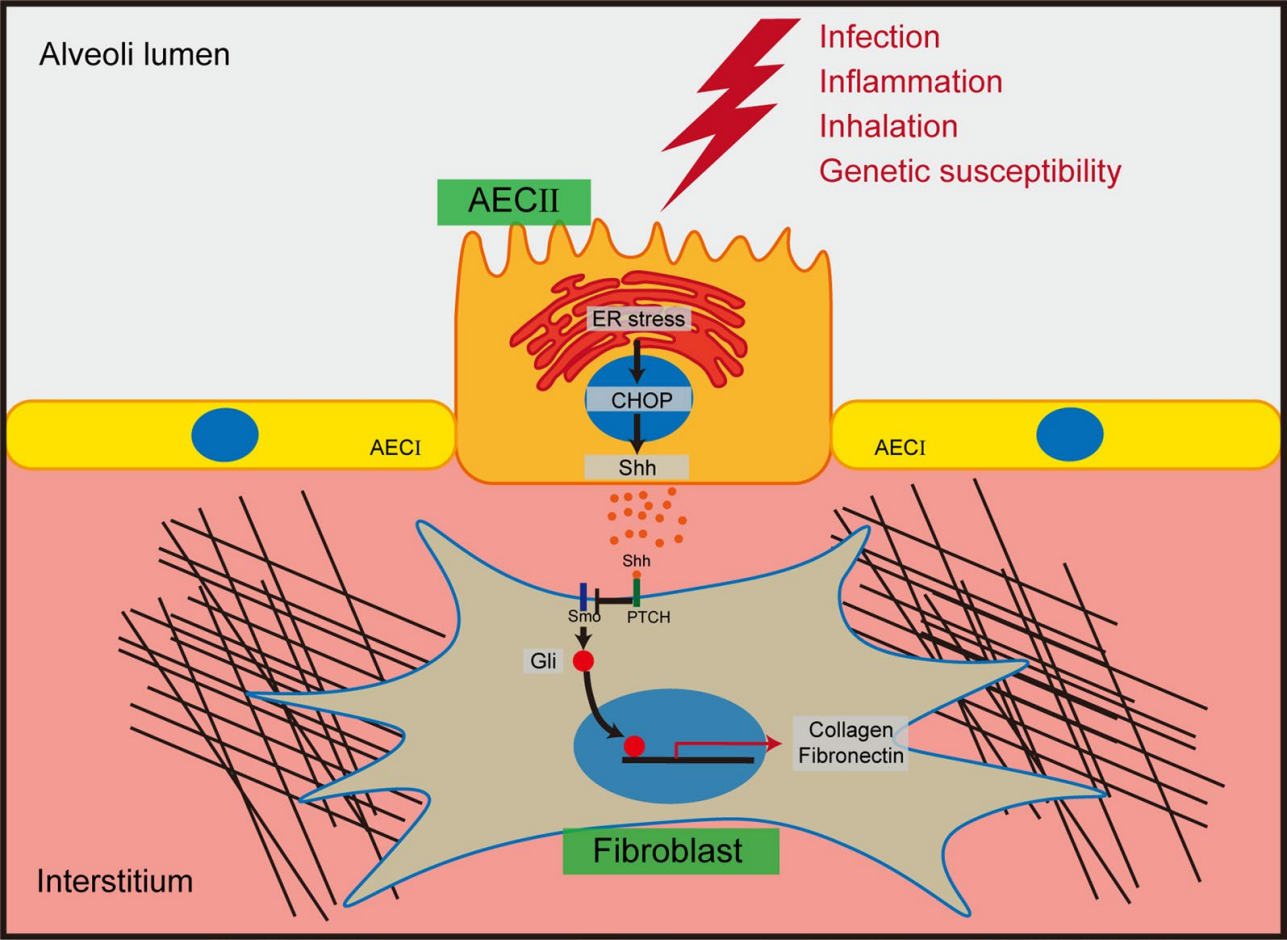
Schematic model of the CHOP induced Shh secretion by AECII and activation of fibroblast during IPF. Multiple factors contribute to ER stress of AECII during IPF. The UPR induced by ER stress upregulates CHOP. Then, CHOP regulates the expression and secretion in an undefined mechanism. Secreted Shh activates HH signaling of fibroblast and promotes ECM synthesis, thus accelerates the development of pulmonary fibrosis

In summary, our work reveals a link between ER stress and sonic HH signaling pathways during IPF (Fig. 8). AECII suffering ER stress upregulates Shh expression and improves Shh secretion, subsequently activates fibroblasts by HH signaling pathway and is involved in lung fibrosis progression. We also found that CHOP of AECII regulates this process, which can be used as an intervention target to relieve pulmonary fibrosis. However, the molecular mechanism of regulation of Shh by CHOP requires further exploration.

## Supplementary Information

**Additional file 1:** **Table S1**. Characteristics of patients who provided surgical samples. **Table S2**. Sequences of shRNA. **Table S3**. Primers for qPCR. **Figure S1**. Identification of primary human type 2 alveolar epithelial cells (AECII). **Figure S2**. The intervention efficiency of sh*CHOP* for the CHOP expression in AECII. **Figure S3**. IPF lung show destructed alveolar structure and ECM accumulation. **Figure S4.** Gross expression levels of key factors in the lung. **Figure S5**. MTT assay for tunicamycin treated AECII.

## Data Availability

Original data can be requested from the corresponding author.

## Abbreviations

CHOP: C/EBP homologous protein
Shh: Sonic Hedgehog
GLI: Glioma-associated oncogene homolog
ER: Endoplasmic reticulum
AECII: Type 2 alveolar epithelial cell
IPF: Idiopathic pulmonary fibrosis
TM: Tunicamycin
shRNA: Short hairpin RNA
CM: Conditioned medium
qPCR: Quantitative chain reaction
pro-SPC: Prosurfactant protein C
