# Supplementary material for: C/EBP homologous protein promotes Sonic Hedgehog secretion from type II alveolar epithelial cells and activates Hedgehog signaling pathway of fibroblast in pulmonary fibrosis
Source: Respir Res. 2022 Apr 8;23:86. doi: 10.1186/s12931-022-02012-x (PMC8991723; doi:10.1186/s12931-022-02012-x)
Supplement: Supplementary file 1 — Additional file 1: Table S1. Characteristics of patients who provided surgical samples. Table S2. Sequences of shRNA. Table S3. Primers for qPCR. Figure S1. Identification of primary human type 2 alveolar epithelial cells (AECII). Figure S2. The intervention efficiency of shCHOP for the CHOP expression in AECII. Figure S3. IPF lung show destructed alveolar structure and ECM accumulation. Figure S4. Gross expression levels of key factors in the lung. Figure S5. MTT assay for tunicamycin treated AECII. [file 12931_2022_2012_MOESM1_ESM.docx]

**Supplemental Material**

**C/EBP homologous protein promotes Sonic hedgehog secretion from type Ⅱ alveolar epithelial cells and activates Hedgehog signaling pathway of fibroblast in pulmonary fibrosis**

Xiaoyu Yang^1^, Wei Sun^1,2^, Xiaoyan Jing^1^, Qian Zhang^1^, Hui Huang^1^, Zuojun Xu^1^

1 Department of Respiratory and Critical Medicine, Peking Union Medical College Hospital, Chinese Academy of Medical Sciences and Peking Union Medical College, Beijing, China.

2 Medical Research Center, Peking Union Medical College Hospital, Chinese Academy of Medical Sciences and Peking Union Medical College, Beijing, China.

**Corresponding authors:** Zuojun Xu

E-mail address: xuzjdoc@163.com

**Supplemental Table 1**. Characteristics of patients who provided surgical samples

|  | Normal | IPF | *P* |
| --- | --- | --- | --- |
| Number | 6 | 6 | - |
| Gender (M/F) | 4/2 | 5/1 | 0.505^*^ |
| Age (year) | 65.3±13.7 | 73.7±6.5 | 0.205^†^ |
| Body mass index (kg/m^2^) | 26.62±6.8 | 24.26±10.5 | 0.657^†^ |
| Smoker (n) | 4 | 3 | 0.558^*^ |
| Pack-years of smoker | 54.25±9.251 | 49.33±4.041 | 0.436^†^ |
| FEV1, % ref | 93.72±4.653 | 85.07±5.286 | 0.013^†^ |
| FEV1/FVC, % | 91.82±3.604 | 85.11±5.341 | 0.029^†^ |
| DLco, % ref | 92.13±2.021 | 36.70±5.874 | <0.0001^†^ |
| Clinical history (%) |  |  |  |
| NSCLC | 83.3 | 0 | 0.0034^*^ |
| SCLC | 16.7 | 0 | 0.2963^*^ |
| Radiological findings (%) |  |  |  |
| Honeycomb | 0 | 100 | 0.0005^*^ |
| Reticular opacities | 0 | 100 | 0.0005^*^ |
| Traction bronchiectasis | 0 | 100 | 0.0005^*^ |
| Ground glass | 50.0 | 16.7 | 0.2207^*^ |
| Consolidation | 83.3 | 33.3 | 0.0790^*^ |

*. The chi-square test was used to compare the differences in composition ratio; †. Values are shown as mean ± SD. Comparison of mean value between two groups using two-tailed *t*-test. The statistically significant difference when *p*＜0.05. FEV1, forced expiratory volume in 1 second; FVC, forced vital capacity; DLco, carbon monoxide diffusion capacity; NSCLC, non-small cell lung cancer.

**Supplemental Table 2**. Sequence of shRNA

|  | Sequence (5’-3’) |
| --- | --- |
| NC | UUCUCCGAACGUGUCACGUTT (sense) |
|  | ACGUGACACGUUCGGAGAATT (antisense) |
| *CHOP* (Human) | GAGCUCUGAUUGACCGAAUTT (sense) |
|  | AUUCGGUCAAUCAGAGCUCTT (antisense) |
| *Chop* (Mouse) | AGCGGAAAGUGGCACAGCUTT (sense) |
|  | AGCUGUGCCACUUUCCGCUTT (antisense) |

**Supplemental Table 3**. Primers for qPCR

| Target Gene | Sequence (5’-3’) |
| --- | --- |
| SHH | CTCGCTGCTGGTATGCTCG |
|  | ATCGCTCGGAGTTTCTGGAGA |
| DDIT3 | ACTCTCCAGATTCCAGTCAGAG |
|  | GCCTCTACTTCCCTGGTCAG |
| GLI1 | AGCGTGAGCCTGAATCTGTG |
|  | CAGCATGTACTGGGCTTTGAA |
| GLI2 | CTGCCTCCGAGAAGCAAGAAG |
|  | GCATGGAATGGTGGCAAGAG |
| PTCH1 | CCAGAAAGTATATGCACTGGCA |
|  | GTGCTCGTACATTTGCTTGGG |
| COL1A1 | GAGGGCCAAGACGAAGACATC |
|  | CAGATCACGTCATCGCACAAC |
| ACTA2 | GTGTTGCCCCTGAAGAGCAT |
|  | GCTGGGACATTGAAAGTCTCA |
| FN1 | AGGAAGCCGAGGTTTTAACTG |
|  | AGGACGCTCATAAGTGTCACC |
| ACTB | ACCCTGAAGTACCCCATCG |
|  | CAGCCTGGATAGCAACGT |
| Shh | TGATGACTCAGAGGTGCAAAG |
|  | GGTCACTCGCAGCTTCACT |
| Ddit3 | CCTAGCTTGGCTGACAGAG |
|  | GTCAGGCGGTCGATTTCC |
| Actb | CTACAGCTTCACCACCACAG |
|  | CTACAGCTTCACCACCACAG |

**
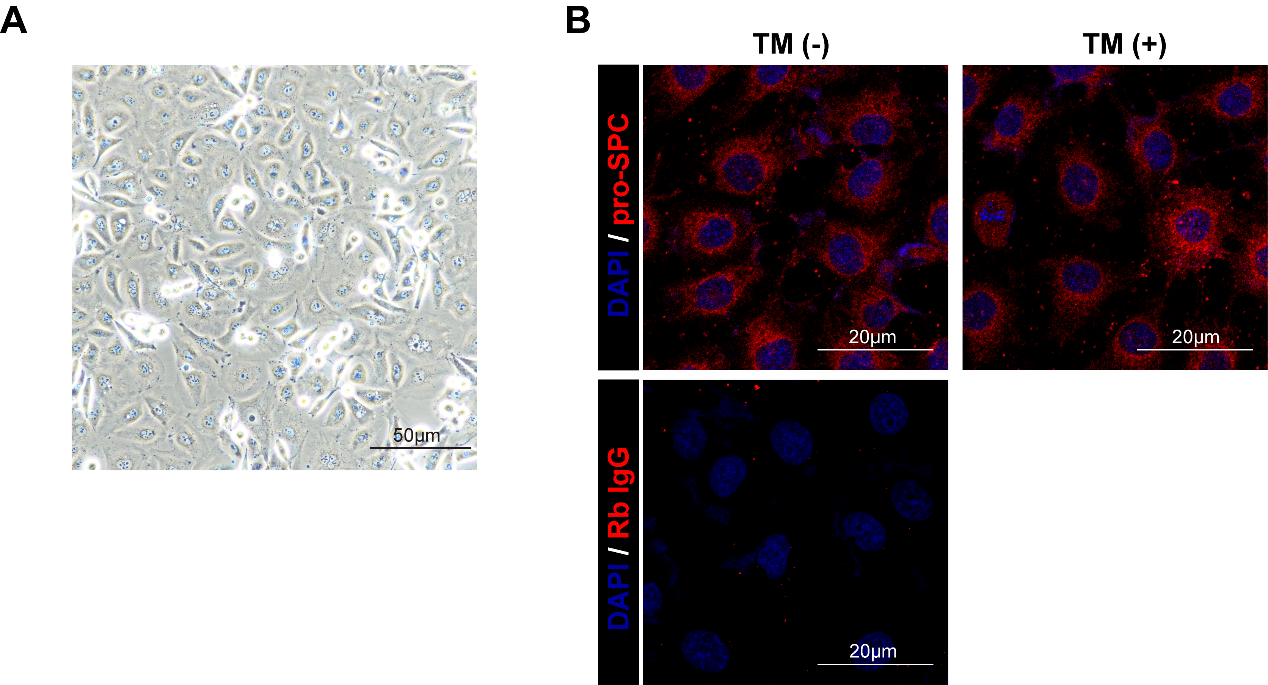
Figure S1. Identification of primary human type 2 alveolar epithelial cells (AECⅡ). (**A) Confluent grown AECⅡ under phase-contrast microscopy. (B) The expression of pro-surfactant protein C (pro-SPC) of AECⅡ with TM free and TM(1μg/mL) treatment. TM, tunicamycin.

**
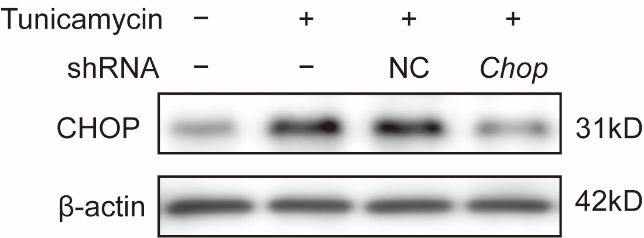
**

**Figure S2. The intervention efficiency of sh*Chop* for the CHOP expression in AECⅡ.** shCHOP blocked tunicamycin-induced CHOP expression in AECⅡ. Scrambled shRNA (NC) acts as control.

**
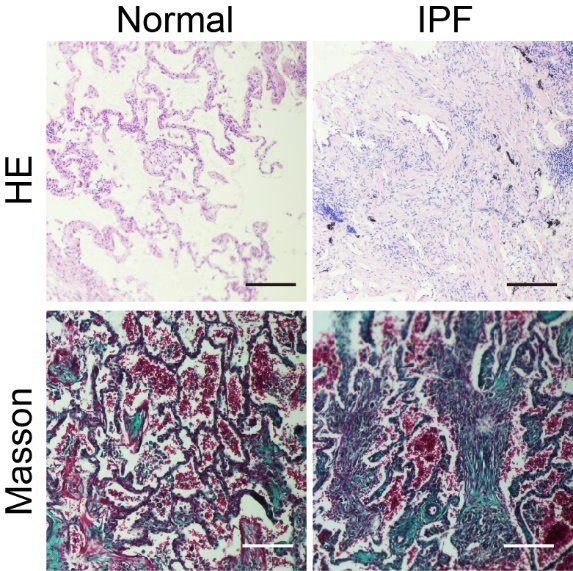
**

**Figure S3. IPF lung show destructed alveolar structure and ECM accumulation.** HE and Masson’s trichrome staining confirmed the diagnosis of all selected IPF lung tissue. Bar=100μm.


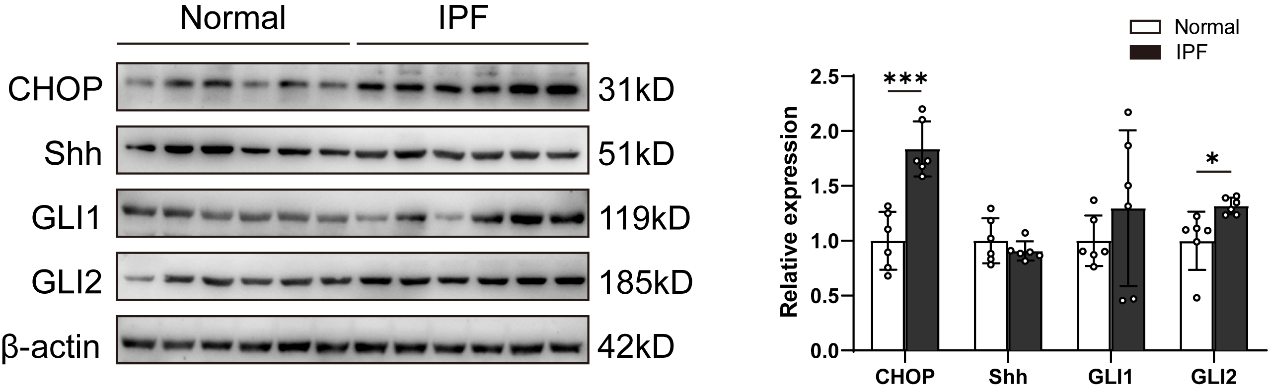


**Figure S4. Gross expression levels of key factors in the lung.** The expression of CHOP, Shh, and GLI1/2 in the non-fibrotic (Normal, n=6) and IPF (n=6) lungs was evaluated by Western blot. Quantification of expression was normalized to the mean grey value of the normal group.


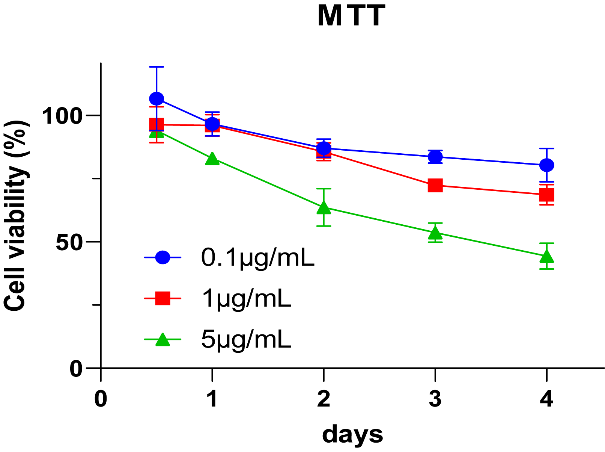


**Figure S5. MTT assay for tunicamycin treated AECⅡ.**
